# Assisted suicide for prisoners: An ethical and legal analysis from the Swiss context

**DOI:** 10.1111/bioe.13005

**Published:** 2022-01-18

**Authors:** Yoann Della Croce

**Affiliations:** ^1^ Department of Political Science and International Relations University of Geneva Geneva Switzerland

**Keywords:** assisted suicide, autonomy, death, euthanasia, prison, rights, Switzerland

## Abstract

Should prisoners be allowed to access assisted suicide? Whereas the ethical and legal issues regarding assisted suicide have now been extensively discussed in the literature, surprisingly scarce attention has been given to the pressing issue of inmates requesting assistance in dying. Through an analysis supported by the Swiss legal framework, I first argue that the principle of equivalence in prison medicine, which states that prisoners ought to receive the same level of health care as the general population does not prove a solid basis in arguing for prisoners’ right to assisted suicide. Over the course of the paper, I defend the view that the right to access assisted suicide is to be understood as a liberty that cannot be removed from incarcerated individuals. I argue that removing such a liberty cannot be consistently held within a legal framework where the death penalty does not exist, for doing so necessarily forgoes the State's ability to decide on when and how prisoners' lives end, in turn necessarily leaving them the liberty to end theirs when they decide so. I finally argue against the position that the capacity for autonomous choice is lacking in inmates by disentangling the particular features of the prison‐setting and show that the context of incarceration is not so substantially different from regular cases of suicide assistance that it warrants a difference in treatment. The position I propose proves important in order to both respect prisoners' rights and ensure they retain a minimum level of control over their existences.

## 1 INTRODUCTION

Should prisoners be allowed to access assisted suicide? Whereas the ethical and legal issues regarding assisted suicide have now been extensively discussed in the literature, surprisingly scarce attention has been given to the pressing issue of inmates requesting assistance in dying. In 2015, the case of Frank van den Bleeken, a Belgian inmate who requested assistance in suicide because the institution he was detained at consistently failed to provide him with the psychiatric care he required, sparked a discussion regarding whether or not prisoners should be allowed to access the procedure.^1^ Similarly, in 2018 a Swiss inmate made a request to right‐to‐die society EXIT because of an incurable lung disease and mental illness, arguing that refusing him access to assistance constituted psychological torture. Competent judicial authorities have clearly stated that so far there exists no clear guidelines regarding prisoners' requests, however emphasizing the fact that there will be no way that assisted suicide could be used as an escape from the prison sentence.^2^ The issue is moreover becoming more and more timely, knowing that prison populations are steadily ageing, making death in prison not just an anecdotical event but a concrete reality that legislators, policymakers and bioethicists ought to take seriously.^3^ So far, no jurisdiction that allows assisted suicide has explicitly addressed the issue at stake here, with the exception of Canada where it has been deemed rightful.^4^

The objections against assisted suicide for inmates are manifold. First, some have argued that the detention context may hinder requestors' autonomy and thus worry that the extent to which their choice is truly free is compromised, at least for cases where suffering is directly caused by the incarceration.^5^ In this sense, they do not object to the practice in principle, but rather worry about potential dangers endemic to the prison setting. Second, others have raised the concern that allowing so may be too reminiscent of the death penalty or worse, may allow for reintroducing the death penalty in disguise.^6^ Finally, there is the concern raised by the aforementioned Swiss judicial authorities that assisted suicide may be considered as an evasion from the sentence requestors are serving. I leave this latter issue aside for it has been argued elsewhere that such a claim ultimately proves to be untenable in a democratic liberal context in addition to being only sustainable if one endorses an extreme retributivist philosophy of punishment, which does not reflect the contemporary Swiss legal landscape.^7^ On the other side of the debate, justifications for granting inmates access to assisted suicide are based on the legal principle of equivalence of care in prison medicine, which states that prison health authorities ought to provide the same level of care that is available for the general population to the incarcerated population.^8^ The principle of equivalence, while being of legal nature, has shown to have significant normative traction in the field of medical ethics.^9^ The ethical questions that arise in the present case thus tackle different analytical levels: Is it wrong, *concerning the penal system*, to allow (or forbid) inmates to be assisted in suicide? And is it wrong, *concerning inmates*, to allow (or forbid) them to be assisted in suicide? Answering these questions requires one to distinguish the justification of a principle (for instance the principles that support the penal system) from the justification of particular practices that fall under this principle (in this case, inmates' suicide assistances).^10^ Both raise different normative difficulties, and in order to propose a comprehensive argument on the matter, one must be careful to address them separately.

I first argue that in the Swiss legal context the principle of equivalence is not enough to justify prisoners' liberty to access suicide, and thus defending this position requires a different justification. I offer an argument for such a justification by demonstrating that there is nothing in inmates' statuses or situations that could warrant a difference in treatment regarding access to suicide assistance. I do so by arguing against the idea that assisted suicide can be understood as a concealed form of the death penalty. Quite the opposite, I argue that forbidding assisted suicide is incompatible with the contemporary Swiss penal system, and more broadly with any jurisdiction's penal system that has abolished the death penalty. This is because one of the essential features of the death penalty is the ability to effectively end one's life in a time and manner decided by the competent authority, thus overriding one's ability to choose for themself. Thus, jurisdictions that do not have the option of the death penalty do not have the ability to exert a power of decision over the timing and manner of the convict's death. Doing so nonetheless would in fact result in the State making use of means that rely on the idea of the death penalty, and as such forbidding assisted suicide for prisoners would in fact be a form of concealed death penalty. I then show that the carceral setting is not as relevant as it seems when evaluating how autonomous their decision to die is. This is not to say that it is irrelevant, but rather that it is not sufficiently different from “standard” cases of assisted suicide to limit access, even in the hard case of “existential” suffering directly caused by the incarceration. I then conclude on some remarks that argue for the equal moral worth of prisoners and how denying them access constitutes a serious moral and political injustice.

## 2 THE LIMITS OF THE PRINCIPLE OF EQUIVALENCE IN THE SWISS CONTEXT

Many advocates for prisoners' right to access assisted suicide have defended their position by arguing for the principle of equivalence of care (hereafter referred to as the principle of equivalence). According to the principle of equivalence, it must be guaranteed that prison populations have access to the same level of health care that is available to the general population.^11^ As prisoners are put in detention by the State, this access must be guaranteed by the State for they have a positive obligation of care towards them. Canadian legal authorities have recognized this obligation after the implementation of Bill C‐14 in 2016, which establishes the legal framework for assisted suicide, or medical assistance in dying (MAiD). This legislation, combined with Section 86 of the Corrections and Conditional Release Act, which guarantees essential health care to inmates, constitutes a guarantee that prisoners can effectively access MAiD. This is due to the fact that MAiD qualifies as health care in the Canadian context: it is performed by medical professionals and does not differ from other health services in its funding from official authorities.^12^

It is however unclear how well this argument translates into the Swiss context. Switzerland has a unique legislation when it comes to assisted suicide, for it possesses no clear legal statute that frames the practice, aside from Art. 115 of the Swiss Penal Code, which allows for suicide assistance as long as it is not performed for selfish motives. Assisting suicide for altruistic reasons is thus lawful. There is in fact no obligation for the act to be lawful that it be performed by a physician or a medical professional.^13^ An interesting feature of this legal context is that it does not identify a specific type of suffering one ought to be enduring in order to qualify for assistance; access is thus not limited to terminal illnesses. In practice, right‐to‐die societies such as EXIT or Dignitas facilitate the process for requestors and may help them find a physician willing to assess their decision‐making capacity and prescribe the lethal drugs used for the suicide.^14^ Recent research has shown that the “right to die” in Switzerland is best understood as a liberty or privilege, which does not give rise to an obligation from the State aside from one of non‐interference.^15^

Now, how does the principle of equivalence fall within the Swiss framework for assisted suicide? The problem does not lie with the principle of equivalence itself, for it is evident that inmates are only deprived of their right to move freely and there is no reason to deprive them of access to health care. Such a deprivation would in fact constitute a violation of Art. 3 of the European Human Rights Convention (hereafter simply referred to as the Convention), which protects individuals against torture or inhumane treatment.^16^ Swiss inmates are effectively entitled to the same level of health care as any other Swiss citizen. The problem lies with the extent to which the principle applies to assisted suicide. Indeed, in order for such a justification to hold ground, it must first be demonstrated that suicide assistance in the Swiss context actually qualifies as health care. This proves to be a difficult endeavor. I propose to test the Swiss case against two different definitions of care, the first being context‐dependent and the second being strictly philosophical. Looking back at the reasons invoked by Downie, Iftene and Steeves, it is understood that in order to qualify as health care, a medical procedure ought to be, at least, performed by medical professionals and its costs are to be covered by official instances through State funding. The Swiss context as it has been outlined above does not meet either of these two criteria, and therefore suicide assistance cannot be considered as care according to that metric. Norman Daniels provides us with a seminal definition of the term, according to which “care” encompasses all actions that ensure the normal functioning of a given individual.^17^ One could make a long‐shot argument and claim that assisted suicide can ensure the halt of abnormal functioning for a given individual, but even claiming so falls short of the definition. It cannot reasonably be said that alleviating of suffering that results in death actually ensures the normal functioning of the deceased individual. According to both of these definitions, it is hard to see how assisted suicide, in Switzerland, qualifies as care and thus should be understood to be covered by the principle of equivalence. The Swiss Academy of Medical Sciences has furthermore insisted on multiple instances through the issuance of medical‐ethical guidelines that assisted suicide is not in itself health care and does not fall within the scope of a physician's duties, even though it is usually in fact performed by a physician, hence the need for the existence of guidelines.^18^

## 3 INMATES, RIGHTS AND INTERFERENCE

Having established that access to assisted suicide cannot be guaranteed by the principle of equivalence allows us to frame the issue in a different manner. Since assisted suicide is a liberty enjoyed by Swiss citizens, and since Swiss inmates remain Swiss citizens, it would certainly appear that in virtue of their equal moral standing they should be guaranteed equal concern and access to the same liberties as the general population. It is *non sequitur* to state that a change in legal status automatically implies a change in moral status; equality of status remains a core value to be owed to all citizens, regardless of them being convicted of a crime or not.^19^ That is, of course, insofar as the exercise of this liberty does not depend on freedom of movement, which is the right meant to be suspended through incarceration. Indeed, according to Art. 74 of the Swiss Penal Code, restrictions on the exercise of rights is only justified insofar as it is required by the deprivation of liberty of movement. However, things become complex when we consider that deprivation of freedom of movement implies, as a corollary, deprivations of the liberties that explicitly rely on free movement. For instance, an inmate *de facto* loses her liberty to drive her car while she is being detained, even though the car remains in her possession: the nature of incarceration makes it impossible for her to access her vehicle and she thus loses the ability to enjoy its use. These corollary restrictions are widely understood to be unproblematic, and they are furthermore consistent with the content of Art. 74. The question we are thus left with is the following: Is a restriction of the liberty to access assisted suicide acceptable in virtue of it being a corollary to freedom of movement? Or, to frame it in a different manner, can the penal system justifiably suspend the exercise of this liberty for prisoners as necessary for a sentence's proper serving?

In order to give an answer to these questions, it is crucial to understand what the relationship between the liberty to be assisted in suicide and fundamental rights is; it makes intuitive sense to believe that there is something more important lying behind suicide assistance than behind driving one's car. This has furthermore been highlighted by doctrine produced by the European Court of Human Rights (ECHR) in two significant cases that have marked legal precedents in the Swiss legal framework. In both rulings of *Haas v. Switzerland* 20 (§51) and *Gross v. Switzerland* 21 (§59), the Court recognized that the right to decide on how and when one's life will end, assuming he or she possesses the capacity to freely reach a decision on this matter, falls within the scope of the right to private life, which is protected by Art. 8 of the Convention. This right to private life is also protected by Art. 13 of the Swiss Constitution, and it is not to be suspended through incarceration.^22^ Incarceration is meant only to deprive citizens of one right, that of freedom of movement, and inmates' right to private life is thus protected by both the Constitution and the Convention. It would therefore appear at first glance that there is no legal ground to deprive prisoners of their liberty to access assistance in dying. This reading is however simplistic, for the Convention allows interference with the right to private life in certain specific cases. Art. 3 al. 2 of the Convention reads as follows:

There shall be no interference by a public authority with the exercise of this right except such as *in accordance with the law* and is necessary in a democratic society in the interests of national security, public safety or the economic well‐being of the country, for the prevention of disorder or crime, for the protection of health or morals, or *for the protection of the rights and freedoms of others*.^23^ (My emphasis)

It is worth emphasizing that both of the aforementioned conditions must be met for interference to be justifiable; it must be (a) lawful and (b) necessary for one of the interests listed above. In order to make a solid case for the defense of prisoners' liberty to access assisted suicide, it is necessary to address both of these conditions and show that neither of them provides sufficient ground for interference. In what follows, I argue that (a) cannot be fulfilled because of the incompatibility between denying access to assisted suicide for prisoners and the abolition of the death penalty. I then argue that regarding (b), the only relevant dimension is the ultimate one of the Convention's third article, namely the protection of the rights and freedoms of others. This final dimension has to do with the standard concern associated with the legalization of assisted suicide, namely the so‐called “slippery slope.” In carceral settings, one could argue that conditions for autonomous decision‐making are so inimical that allowing for access may hurt the class of prisoners as a whole. I argue that none of these arguments carries enough weight to warrant interference with the prisoners' liberty.

Before turning to the issues regarding the potential for a concealed death penalty and the difficulty of autonomous decision‐making, I shall close this section on rights and interference with a few words on the idea of compassionate release. Indeed, the Council of Europe has issued recommendations that terminally ill prisoners should be able to be released from prison in order to die outside, regardless of the danger presented by the prisoner (who in this case may be allowed to leave prison but remain under supervision). However, Shaw and Elger have pointed out two major flaws in how compassionate release interfaces with assisted suicide in Switzerland: first, prisoners released on compassionate grounds are often transferred to hospitals or homes where assisted suicide is not necessarily performed (some hospitals in effect explicitly refuse to have assisted suicide performed on their premises) and second, the criteria for eligibility to compassionate release may be stricter than those of assisted suicide, leaving prisoners unable to exercise their liberty because of administrative mismatch between conditions.^24^ As such, while the possibility of compassionate release in order to access suicide assistance is indeed legally available to Swiss inmates (in the sense that it is at least not explicitly forbidden), the lack of guidelines surrounding the specific ethical issue of assisted suicide for prisoners creates an administrative and legal blur that effectively prevents them from accessing it.

## 4 ASSISTED SUICIDE AS DISGUISED DEATH PENALTY

An objection that has been raised against assisted suicide for prisoners is that it may seem too reminiscent of capital punishment^25^ or worst, that it may in effect be a concealed version of the death penalty.^26^ The logic behind these arguments is fairly straightforward. By allowing access to assisted suicide, the State indirectly sends the message that it is acceptable to die behind bars, and more important, it may seem to acknowledge that some sentences are so harsh that it is rational and justifiable to ask for the ending of one's life in order not to go through the suffering of prison. To put it bluntly, one could sum it up as a crude “Kill yourself” injunction from the State to its detainees. Since the State has a positive obligation of responsibility towards inmates and since citizens, especially vulnerable ones, have a claim‐right to be protected from irrational suicide, as highlighted by *Haas v. Switzerland* (§54), then it is at odds with Swiss law and should thus not be condoned. This argument however misses the mark and is ultimately concerned with the issue of whether or not prisoners are truly capable of making an autonomous choice regarding the voluntary termination of their lives, which I will address later. The focal point of the argument is not, *in fine*, the legal framework itself, but rather the subjects of this framework.

There is a more abstract normative idea at play here that is much more relevant to the pressing question of whether or not it is lawful to forbid inmates from accessing suicide assistance: the abolition of the death penalty. Since 1942 and per Art. 10 al. 1 of the Swiss Constitution, it is made very clear that capital punishment is strictly forbidden and must never be applied as a mean of punishment. This is furthermore highlighted by the clear fact that the suffering engendered by punishment is meant only to be caused by the deprivation of freedom of movement and strictly nothing more.^27^ Substantially, the abolition of the death penalty implies that the State has no power over the death of prisoners in its array of means of punishment. It simply cannot, and must not, incorporate features that include the deaths of prisoners in their punishment.

This is however exactly what it does if suicide assistance is denied to prisoners. By forbidding access, the State reintroduces the language of death in the *conditions* of punishment. The moral validity of the intention is of no interest here, what truly matters is the consequence of such a legislation. Denying access expresses that it is in effect acceptable for the State to exercise power over the deaths of some of its citizens, regardless of what they wish for themselves. It effectively exerts control over the deaths of inmates and necessarily becomes a part of the framework in which punishment is defined, therefore being incompatible with the absence of the death sentence. There is substantially no significant difference between setting a time and manner for a prisoner's death and refusing to let him or her set a time and manner for himself or herself, no matter what the reasons for refusal are, that is as far as one is concerned with what relations of power and control are displayed between the State and its citizens. Therefore, claiming that allowing prisoners to be assisted in dying is tantamount to a concealed reintroduction of the death penalty cannot be consistently defended for a refusal of access relies on tacit principles that ought to be excluded from punishment in an abolitionist legal framework. In fact, forbidding inmates from being assisted in suicide in the context of an abolitionist framework may even be argued to be in itself a form of concealed death penalty, since in such a framework, as I have argued, the State does not possess a power of control that reaches out to how and when prisoners may end their lives. While it is true that the State does have a duty to ensure that prisoners do not die during their time in prison, it cannot be argued that this duty may encompass the prohibition of assisted suicide for prisoners since this duty arises from the fact that, while incarcerated, inmates are under the direct responsibility of the State, which must in turn ensure their safety (i.e., not dying or being harmed). The only case where one may argue that assisted suicide ought to be prohibited on the ground of safety is if the capacity for autonomous decision‐making may be jeopardized or absent, the issue that I turn to in the next section.

## 5 AUTONOMY AND THE PROTECTION OF RIGHTS AND FREEDOMS

Having established that the legal conditions for refusing access to suicide assistance for inmates must necessarily rely on principles that cannot be dissociated from the idea of the death penalty, thus being unlawful in the context of Swiss law but also of all other jurisdictions who share this abolitionist characteristic, I turn to the issue of autonomy. The line of argument based on autonomy that supports denial of access to assisted suicide for prisoners is fairly straightforward and is in substance similar to the one given for any vulnerable group requesting aid in dying. According to this argument, the prison setting is so unsuitable for autonomous and freely made decisions that an inmate's request to die cannot be categorically taken to be well considered or persistent, for if circumstances were different (if the prisoner was not in prison), then it is unlikely that some requestors would make the same demand. This is in essence the same idea that lies behind arguments against physician‐assisted suicide for people with disabilities, for their particular situation of increased social vulnerability would seemingly make an autonomous choice virtually impossible.^28^ There is, furthermore, the risk of coercion by other inmates or wardens, who could manipulate the requestor and mislead them into believing their life is not worth living anymore, for malicious intent. For these reasons, it would thus be best to stay the hand in the case of prisoners. I argue against both of these claims.

It is however important to nuance the argument, for it makes a difference between two distinct kinds of requestors amongst prisoners. The first kind contains those inmates who are terminally ill and request to be assisted because their suffering has become too great and their end is near. Just as it is the case with the general population, these cases are considered unproblematic and it is hard to imagine an argument that would specifically forbidding them from undergoing the procedure; doing so would most likely even constitute a violation of Art. 3 of the Convention. The second kind is much more controversial and is concerned with what has been called “prison tedium,” that is, being “tired of life in prison.”^29^ These detainees are not terminally ill but are suffering of what can be described as “existential” suffering. They will not die from the cause, understood in the medical or physiological sense, of their suffering, they just do not want to go on because they feel their lives are simply not worth living anymore. This has proven to be a point of contention in the literature.^30^ In these cases, suffering ought to be understood as directly imputable to incarceration and not any underlying illness or disease. But is prison tedium really special, as far as the possibility of autonomous choice is concerned? And does it warrant a differentiation of rights‐exercising because of its special features? To the former I argue that it is not, and to the latter that it does not. Consider two hypothetical cases.

Adam, a bright 22‐year‐old college student and soon to be professional basketball player, leaves his university building, off for lunch with his girlfriend. He arrives at a pedestrian crosswalk in order to get across the street to the restaurant he is awaited at. The crossing sign for pedestrians is red, yet no cars seem to be coming. Adam, slightly late, decides to jaywalk across the boulevard. However, Adam did not look out cautiously enough, and soon enough a car rams into him at full speed. He wakes up at the hospital, only to learn that while his days are not endangered, he is now tetraplegic and will most likely never be able to play basketball or walk anymore. His career and dreams are crushed and, after a while, decides that his life is not worth living anymore and requests suicide assistance.

Now consider the case of Bianca. Bianca is the same age as Adam and attends the same college. She lives in an old apartment where the sound insulation is terrible. Exhausted by the constant noise made by her upstairs neighbors, she one day decides that she has had enough and schedules a visit on Friday evening after class to make sure her neighbors remain forever quiet. Friday evening comes, and she proceeds to grab the biggest knife in her kitchen, walks to her neighbors' door and, in cold‐blood, brutally murders them. Bianca is then caught by the police and sentenced to 25 years in prison for her wrongdoings. While behind bars, she decides that her life is not worth living for the better part of her youth will be spent in prison and it will be impossible for her to ever get back to a normal life after she has served her sentence. She furthermore cannot cope with the harsh reality of prison life. It is also worth stressing that she does not feel any kind of remorse towards what she did; her suffering is directly and only caused by her deprivation of liberty. She requests suicide assistance.^31^

It is fair to say that Adam's case would not be considered as problematic whereas Bianca's case would most certainly be. However, the differences between these two cases are thin, at least when it comes to ethical decision‐making. Both have performed an act, *X*, which was performed with a characteristic, *Y*, which denotes a specific intention. They both could have done otherwise. Because of *X*, both end up in a situation *Z* that radically alters the courses of their respective lives. What differs is that in Bianca's case, *X* has the additional feature of *actus reus* (a wrongful act) and *Y* has the additional feature of *mens rea* (a guilty mind). *Z* is, in both cases, substantially identical, even though the chain of events that eventually led to its bringing about differs. That is, at least as far as the *capacity* for autonomous choice is concerned. This implies that in order to justify a difference in treatment, justification must be found in either the features of *mens rea* or *actus reus*. However, as I have shown before, doing so necessitates a shift in the philosophy of punishment which cannot be held alongside contemporary abolitionist principles, and is thus not an option. Regarding the degree of autonomy of the choice, there is no reason to assume that Bianca is less autonomous than Adam in her request to die. Both are dealing with extraordinary events that will radically alter the rest of their respective lives. Just like Adam, Bianca (and all prisoners) must be evaluated by an expert in order to determine her competence and how free her choice is. Like all requestors, competence and freedom ought to be presumed and must be confirmed or invalidated by an expert psychiatrist.^32^ If the law grants access to Adam, or at least considers granting it, it must then do the same to Bianca. If one wishes to argue otherwise, the one has to show how the nature of *Z* differs between Bianca's and Adam's situations. Surely, Bianca's actions have rightfully led her to prison, and she is paying the price for her wrongdoings. But arguing that because of the nature of these actions her claims of suffering are not to be taken seriously and somehow less valid than those of the general population is going down a dangerous slippery slope that ultimately leads to the dehumanization of inmates and failure to consider them as equal citizens, which, notwithstanding the content of some populist rhetoric, they remain.

Claiming that prisoners deserve the additional retribution of a miserable death rests on the assumption that they are somehow less valuable members of society or as some have put it, some form of social non‐entity that cannot be the source of valid moral claims.^33^ This is not, and must not, be the case. Prisoners are due equal respect in respect to their suffering and failing to acknowledge the seriousness of their pain on the ground that they have committed criminal acts is tantamount to treating them as second‐class citizens to whom we owe less respect and concern than the law‐abiding citizen. Whether or not inmates are given the respect they are owed is largely dependent on State action for it is the State that is the main provider of status in a society: it distributes basic rights and recognizes them and it influences how citizens view each other.^34^ Some services must be owed to all, and these services include access to the equal exercise of rights; no citizen is less entitled to these goods than others because of a particular feature they hold, no matter if this feature was the result of an intentional action or not. Differentiating treatment and access on these grounds is, in effect, an attack on self‐respect (or self‐esteem).^35^ In the case of prisoners, this not only constitutes additional unwarranted retribution but also unjustified extraordinary punishment, for their dignity must be preserved at all times during their sentence, and this ought to be part of the positive obligations of the State regarding the detainees under its responsibility.

## 6 CONCLUSION

Throughout this paper, I have argued that even though the principle of equivalence does not cover assisted suicide in the case of prisoners, interference with their choice to be aided in ending their lives is still unwarranted. With the help of illustrations from the Swiss context, I have shown that denying access to assisted suicide in virtue of incarceration is incompatible both with the absence of the death penalty and with the principles that regulate detention, contrary to popular belief. I have then argued that regarding the difficulty inmates face in making autonomous choices, there is no substantial difference between the prisoners' situation and other cases like severe disability following an accident. This is not to say that the problem automatically disappears, it is rather intended to mean that the difficulties that arise when considering requests from inmates enduring existential suffering are the same difficulties that are found when considering any demand for assisted suicide that do not find their source in a terminal illness. Therefore, the conditions for interference put forward by Art. 3 of the Convention are absent and refusal cannot be justified. I have translated these legal conditions into the terminology of moral philosophy in order to show that neither on a legal nor moral standing do the objections to assisted suicide for prisoners hold ground. It may even be argued that granting prisoners a wider range of options regarding end‐of‐life practices will in fact increase their perception of choice and subsequent autonomy, which is known to have a positive effect on well‐being.^36^ This might in turn reduce the will to die based on existential suffering in the long‐run. While public debates on the matter are still young and much more has yet to be said, I hope that this contribution will spark conversation on a timely issue that is bound to become more and more prominent as legislations on aid in dying become more liberal. Prisoners, in virtue of their equal moral worth and the validity of their suffering, are owed a thorough assessment of this question and must not, as is unfortunately still too often the case, be left behind in the public debate once again.

## CONFLICT OF INTEREST

The author declares no conflict of interest.

## Footnotes

1 Menétrey‐Savary, A. C. (2015). *Mourir en prison*. Infoprisons. Retrieved March 18, 2020, from www.infoprisons.ch

2 Bern prisoner requests assisted suicide, 2018. Swissinfo. Retrieved March 18, 2020, from https://www.swissinfo.ch/eng/unprecedented-case_swiss-prisoner-requests-assisted-suicide/44286542

3 Handtke, V., Bretschneider, W., Wangmo, T., & Elger, B. (2012). Facing the challenges of an increasingly ageing prison population in Switzerland: In search of ethically acceptable solutions. *Bioethica Forum, 5*(4), 134–141; Handtke, V., & Wangmo, T. (2014). Ageing prisoners' views on death and dying: Contemplating end‐of‐life in prison. *Bioethical Inquiry, 11*, 373–386.

4 Downie, J., Iftene, A., & Steeves, M. (2019). Assisted dying for prison populations: Lessons from and for abroad. *Medical Law International, 9*(2–3), 1–19.

5 Shaw, D., & Elger, B. (2016) Assisted suicide for prisoners? Stakeholder and prisoner perspectives. *Death Studies, 40*(8), 479–495; Hanson, A. (2017). Psychiatry and the dying prisoner. *International Review of Psychiatry, 29*(1), 45–50; Ibid: 16.

6 Willems, A. (2015). *Euthanasia of a detainee: Granting a prisoner's request*. Centre for Crime and Justice Studies. https://doi.org/10.1080/09627251.2015.1026563

7 Della Croce, Y. (2020). The great escape? A liberal perspective on assisted suicide for prisoners. *Ethics, Medicine and Public Health, 15*, 100566.

8 Handtke, V., & Bretschneider, W. (2015). Will I stay or can I go? Assisted suicide in prison. *Journal of Public Health Policy, 36*(1), 67–72; Shaw & Elger, op. cit. note 5, p. 488; Downie et al., op. cit. note 4, p. 6.

9 Niveau, G. (2007). Relevance and limits of the principle of “Equivalence of Care” in prison medicine. *Journal of Medical Ethics, 33*(10), 610–613.

10 This methodology is directly drawn from Rawls, J. (1955). Two concepts of rules. *Philosophical Review, 64*(1), 3–32.

11 Charles, A., & Draper, H. (2012). Equivalence of care in prison medicine: Is equivalence of process the right measure of equity*? Journal of Medical Ethics, 38*(4), 215–218.

12 Downie et al., op. cit. note 4, pp. 5–6; It is worth adding that considering assisted suicide as care does not commit one to classifying it as healing. Against the view that assisted suicide constitutes healing, see Raus, K., Sterckx, S., & Mortier, F. (2011). Is continuous sedation at the end of life an ethically preferable alternative to physician‐assisted suicide? *The American Journal of Bioethics, 11*(6), 32–40.

13 Hurst, S., & Mauron, A. (2003). Assisted suicide and euthanasia in Switzerland: Allowing a role for non‐physicians. *British Medical Journal, 326*(271), 271–273.

14 Ziegler, S., & Bosshard, G. (2007). Role of non‐governmental organisations in physician assisted suicide. *British Medical Journal, 334*(295), 295–298.

15 Hurst, S., & Mauron, A. (2017). Assisted suicide in Switzerland: Clarifying liberties and claims. *Bioethics, 31*(3), 199–208.

16 Andorno, R., Shaw, D., & Elger, B. (2015). Protecting prisoners' autonomy with advance directives: Ethical dilemmas and policy issues. *Medicine, Health Care and Philosophy, 18*, 33–39; Such violations have in effect already been condemned by the European Court of Human Rights, for instance in the *Renolde v. France* ruling where France was found to have failed to comply with their obligation of care by not providing treatment to an inmate suffering from severe mental illness, ultimately leading to his suicide. *Renolde v. France*, 5608/05 (2008).

17 Daniels, N. (2001). Justice, health and healthcare. *The American Journal of Bioethics, 1*(2), 2–26.

18 Swiss Academy of Medical Sciences. (2018). *Management of pain and dying*. Retrieved October 15, 2021, from https://www.samw.ch/en/Publications/Medical-ethical-Guidelines.html. See particularly the following passage “The role of physicians in the management of dying and death involves relieving symptoms and supporting the patient. Their responsibilities do not include offering assisted suicide, nor are they obliged to perform it. Assisted suicide is not a medical action to which patients could claim to be entitled; it is, however, a legally permissible activity” (p. 22). It is worth precising that the scope of this article does not aim at answering the broader question of whether or not assisted suicide *in itself* may qualify as health care. All that my argument aims at doing here is to show that in the Swiss medical–legal context, assisted suicide is uncontroversially admitted to be out of the scope of the procedures that physicians ought to perform.

19 Scanlon, T. (2017). *Why does inequality matter?* Oxford University Press.

20
*Haas v. Switzerland*, 31322/07 (2011).

21
*Gross v. Switzerland*, 67810/10 (2013).

22 Hohl‐Chirazi, C. (2016). *La privation de liberté en procédure pénale suisse: Buts et limites*. Schulthess Editions Romandes.

23 European Convention on Human Rights, Protocol No. 16 (2018). Retrieved March 19, 2020, from https://www.echr.coe.int/Pages/home.aspx?p=basictexts%26c

24 Shaw & Elger, op. cit. note 5, pp. 482–483.

25 Ibid: 484.

26 Willems, op. cit. note 6, p. 16.

27 Brägger, B. F., & Vuille, J. (2012). *Punir, prévenir et resocialiser: De l'arrestation provisoire à la libération conditionnelle*. Stämpfli Editions; Viredaz, B. (2009). *Les principes régissant l'exécution des peines privatives de liberté (art. 74 et 75 al. 1 CP)*. Schulthess Médias Juridiques.

28 Scoccia, D. (2010). Physician‐assisted suicide, disability, and paternalism. *Social Theory and Practice, 36*(3), 479–498; Della Croce, Y. (2020). Taking oppression seriously: Disability and physician‐assisted suicide. *Bioethica Forum, 13*(1), 22–27.

29 Handtke & Wangmo, op. cit. note 3, p. 379.

30 Shaw & Elger, op. cit. note 5, pp. 15–16.

31 Note that this thought experiment aims at bringing to the fore an answer to just the issues of autonomy and rights‐exercising, and as such does not necessarily seek nor need to provide a representative picture of the motivations of prisoners that request suicide assistance. For an in‐depth and accurate account of prisoners' motivations in requesting AS, see Handtke & Wangmo, op. cit. note 3; Shaw & Elger, op. cit. note 5. The argument I seek to make here is that should an extreme case such as Bianca's prove to be unproblematic as far as autonomy and equality of rights‐exercising are concerned, then surely less extreme cases will also prove to be unproblematic with regards to these two specific issues.

32 Andorno et al., op. cit. note 16, pp. 36–37.

33 Cohn, F. (1999). The ethics of end‐to‐life care for prison inmates. *Journal of Law, Medicine and Ethics, 27*(3), 252–259.

34 Porro, C. (2021). Penal disenfranchisement and equality of status. *Journal of Applied Philosophy, 38*(3), 401–414.

35 Scanlon, op. cit. note 19, pp. 26–27.

36 van der Kaap‐Deeder, J., Audanaert, E., Vandevelde, S., Soenens, B., Van Mastrigt, S., Mabbe, E., & Vansteenkiste, M. (2017). Choosing when choices are limited: The role of perceived choice and autonomy in prisoners' well‐being. *Law and Human Behavior, 41*(6), 567–578.

